# Preoperative visualization of the lingual nerve by 3D double-echo steady-state MRI in surgical third molar extraction treatment

**DOI:** 10.1007/s00784-021-04185-z

**Published:** 2021-09-29

**Authors:** Adib Al-Haj Husain, Silvio Valdec, Bernd Stadlinger, Martin Rücker, Marco Piccirelli, Sebastian Winklhofer

**Affiliations:** 1 Clinic of Cranio-Maxillofacial and Oral Surgery, Center of Dental Medicine, University of Zurich, Plattenstrasse 11, 8032, Zurich, Switzerland; 2 Clinic of Cranio-Maxillofacial and Oral Surgery, University Hospital of Zurich, University of Zurich, Rämistrasse 100, 8091, Zurich, Switzerland; 3 Department of Neuroradiology, Clinical Neuroscience Center, University Hospital Zurich, University of Zurich, Frauenklinikstrasse 10, 8091 Zurich, Switzerland

**Keywords:** Anatomy, Lingual nerve, Magnetic resonance imaging, Oral surgery, Radiology

## Abstract

**Objectives:**

To assess the lingual nerve (LN) visualization using a 3D double-echo steady-state MRI sequence (3D-DESS).

**Materials and methods:**

Three readers prospectively evaluated the LN for its continuous visibility in 3D-DESS MRI in 19 patients with an indication for removal of mandibular impacted third molars, using a 5-point scale (4 = excellent to 0 = none). Six LN anatomical intermediate points (IP) were selected and checked for their detectability by a 4-point scale (4 = yes to1 = no). Inter- and intra-rater agreement was evaluated using intraclass correlation coefficient and percentage of agreement.

**Results:**

The average nerve continuity score was 3.3 ± 0.46. In 35% of the cases, the entire course was continuously visible. In 10%, the proximal and 60%, the distal part of the nerve was not continuously visible. Inter- and intra-reader agreement was good (ICC = 0.76, ICC = 0.75). The average detectability score of all IP was 3.7 ± 0.41. From IP1 to IP5, the detectability was excellent; meanwhile, IP6 had lower visibility. The inter- and intra-reader percentage of agreement was 77% and 87%.

**Conclusions:**

The 3D-DESS sequence allowed accurate and continuous visualization of the LN with high reproducibility in more than one-third of the patients. This could improve the preoperative clarification of the LN position and thereby reduce complications during dentoalveolar surgical interventions.

**Clinical relevance:**

3D-DESS MRI might be beneficial in clinical scenarios where the second molar is elongated or presents a difficult rotational position while simultaneously having a close positional relationship to the third molar. Thereby, osteotomy performed more lingually, indicating extended lingual flap detachment may increase the risk of LN damage.

**Supplementary Information:**

The online version contains supplementary material available at 10.1007/s00784-021-04185-z.

## Introduction

Knowing the exact anatomical course of the lingual nerve (LN) is important in various dental and oral surgical interventions. The nerve demonstrates large individual anatomical variability regarding position and proximity to important structures, especially in the third molar region.

Iatrogenic injuries of the LN can occur in various surgical procedures such as osteotomy of mandibular ramus, orthognathic surgery, endodontic treatments, tumor excision, or local anesthetic application prior to extraction of the mandibular third molar and due to the extraction itself. The insertion of dental implants or even general periodontal procedures can also damage the trigeminal nerve’s extracranial branch [1].

Injuring the LN, a rare complication during the removal of mandibular third molars [2, 3], is associated with sensory disturbances in the ipsilateral anterior two-thirds of the tongue [4]. The taste is affected due to the connection with the special sensory taste fibers of the chorda tympani; significantly affected are the fungiform papillae, which represent the largest part of the taste buds and are responsible for the perception of different taste qualities [5, 6]. Besides, it leads to an altered salivary flow rate of the ipsilateral affected sublingual gland [7]. Varying numbers about the frequency of LN damage risk in patients undergoing third molar extraction are given in the literature, ranging from 0.02 to 2% [8]. Reports in the literature suggest that the incidence of surgically induced postoperative temporary LN damage varied from 0 to 37.5%, with permanent LN damage estimated at 0 to 2% [9]. Consequently, most LN injuries recover spontaneously within 8 weeks; meanwhile, long-lasting injuries remain permanent and ensure for affected patients’ significant restrictions in everyday life and loss of quality of life [10].

Panoramic radiography is obtained routinely before third molar extraction. However, in numerous cases, where the roots have close proximity to the inferior alveolar canal with a darkening of at least one of the roots, diversion of the inferior alveolar canal, or where a discontinuous cortical line of the nerve canal is present, two-dimensional imaging is not satisfactory [11]. In these cases, the use of three-dimensional imaging such as cone beam computed tomography (CBCT) is indicated and currently the method of choice to decrease the risk of nerve damage [11]. These conventional x-ray-based imaging modalities can only display nervous tissue indirectly by depicting the osseous boundaries surrounding the nerve canal, whereas the nerve itself cannot be visualized. Magnetic resonance imaging (MRI), with its high soft tissue contrast, is the gold standard for imaging of nervous tissues directly, while it is limited for hard tissue imaging yet. The problem with MRI in the oral cavity is the susceptibility to movement artifacts, complex anatomical courses, small sizes and variations of several blood vessels and nerves, and artifacts due to field inhomogeneity caused by metallic implants or dental restorations [12, 13].

The visualization of the extracranial nerves’ complete course is a major challenge although imaging techniques have greatly improved. Available modalities are high-resolution ultrasound examinations, allowing us to identify the LN intraorally [14], and magnetic resonance neurographic orthopantomogram that allows the simultaneous visualization of bone texture and neural microarchitecture of peripheral nerves [15]. The difficulty in MR neurography has been for a long time the selective and continuous imaging of the thinnest peripheral extracranial nerves. The use of high-resolution three-dimensional turbo spin-echo with variable flip angle (SPACE) STIR sequence with its T2-weighted imaging contrast and background suppression allows depicting the morphology and pathology of peripheral nerves continuously and directly [16].

Other studies, investigating the 3D double-echo steady-state MRI (3D-DESS) sequences showed excellent LN identification [17, 18] and precise localization of the inferior alveolar nerve within the mandibular canal [19]. “Black Bone” MRI sequences—such as 3D-DESS and 3D STIR—showed good feasibility and excellent visualization of the inferior alveolar nerve and LN in healthy subjects, whereby the 3D SPACE STIR showed superior signal to noise ratio and nerve muscle contrast to noise ratio, but on the other hand, the DESS sequence was best suited for the comparability of quantitative LN parameters [18]. DESS combines the FID signal of FISP with the echo-signal of PSIF, increasing its T2* specificity and decreasing signal dropout through dephasing. In this MRI sequence, the mandibular nerve’s peripheral branches, such as the LN, appear as a high signal intensity structure and could be distinguished from adjacent anatomical structures due to the myelin layer surrounding the nerve, allowing excellent visualization by application of the water excitation fat-suppression technique [20]. Fujii et al. focused on the detectability of the medial and distal portion of the LN at the intermediate point (IP) where the LN starts running from laterally to medially, while Burian et al. in 2019 checked the overall LN detectability using multiple MRI sequences. Previously conducted studies had various limitations, such as visualization of the LN in a cohort of patients without an indication for third molar extraction [17] or by comparing the visibility of the LN in MRI without checking for its continuous detectability explicitly in the surgically relevant third molar region [18].

The aim of this study was to investigate the detectability of the entire continuous course and the focal detectability of the intermediate points of the LN in patients with impacted third molars. We focused on the anatomical relationship of the third molar teeth, the anatomic proximity to the alveolar ridge of the lingual cortical plate, and the diameter of the LN using the 3D-DESS sequence.

## Materials and methods

### Study design and setup

Between May 2018 and December 2018, 23 patients were recruited. Four patients did not complete imaging procedures due to not showing up. Therefore, 38 lingual nerves were evaluated (19 patients, each left and right side). The study population included patients with an indication for removal of partially retained, fully retained, or impacted third molar in the third or fourth mandibular quadrant with the additional indication for three-dimensional imaging according to the guidelines of the Swiss association of dentomaxillofacial radiology admitted to the Clinic of Cranio-Maxillofacial and Oral Surgery of the Center of Dental Medicine (University of Zurich) either by a private practitioner or by themselves. MRI data acquisitions were performed by trained neuroradiologists. The subsequent surgical procedure of the mandibular third molar under local anesthesia was performed by oral surgeons. The cohort group included six males (32%) and 13 females (68%); the mean age of the enrolled patients was 30.5 ± 13 years (median age, 25 years; age range, 18–63 years) (Table 1).

**Table 1:** Patient characteristics

Patient characteristics	Total
*N*	19
Gender, male/female, *N*	6/13
Mean (SD) age at scan, years	30.5 (13)
Median age at scan, years	25
Age range, years	18–63
Totally evaluated lingual nerves	38
Clinical indication	Third molar extraction
Retention types [21]	
Type 1, *N*	0
Type 2, *N*	0
Type 3, *N*	11
Type 4, *N*	19
Type 5, *N*	2
Type 6, *N*	0
Type 7, *N*	0
No retention	4
Absent	2

The following criteria were required for the study participants to be included: (1) indication for removal of mandibular partially retained, fully retained, or impacted third molar; (2) male and female patients aged 18 to 65 years. The exclusion criteria were (1) acute odontogenic infection; (2) nerve damage of the inferior alveolar nerve or trigeminal nerve; (3) adjacent implants or metallic reconstructions; (4) pregnancy; and (5) other contraindications for MRI imaging.

The study (BASEC-Nr. 2017-01053) was approved by the the Cantonal Ethics Commission of Zurich (Switzerland). All participants were informed and provided written informed consent for inclusion in the study in accordance with the Declaration of Helsinki and its later revised ethical standards.

Reporting complies with the STROBE (“Strengthening the Reporting of Observational studies in Epidemiology”) guidelines.

### MRI data acquisition

The MR images were acquired with a 3-Tesla Skyra (release VE11c, Siemens Healthineers, Erlangen, Germany) and a Siemens standard 64-channel head-and-neck coil. The used axial 3D-DESS with water excitation MRI sequence had an isotropic acquisition resolution of 0.75 × 0.75 × 0.75 mm^3^ mm together with a receive bandwidth of 355 Hz/Px. The other sequence parameters were field-of-view 242 × 242 × 78 mm^3^; acquisition matrix 320 × 320 × 104; slice oversampling 100%; no parallel acquisition; one signal average; acquisition time 12:24 min:s; TR/TE1/TE2 11.2/4.2/7.7 ms; flip angle 30°; and selective water excitation (Table 2).

**Table 2:** Main technical parameters of the 3D-DESS MRI sequence. The complete protocol is given as supplementary data file

3D-DESS MRI parameters	Total
Acquisition time	12:24 min:s
FOV	242 × 242 × 78 mm^3^
Acq matrix	320 × 320 × 78
Acq voxel	0.75 × 0.75 × 0.75 mm^3^
Number of signal averages	1
TR	11.2 ms
TE1	4.2 ms
TE2	7.7 ms
WFS (pix)/bandwidth (Hz)	1/355
Fat suppression	Selective water excitation
Parallel acquisition	No

### Image analysis

**Fig. 1:**
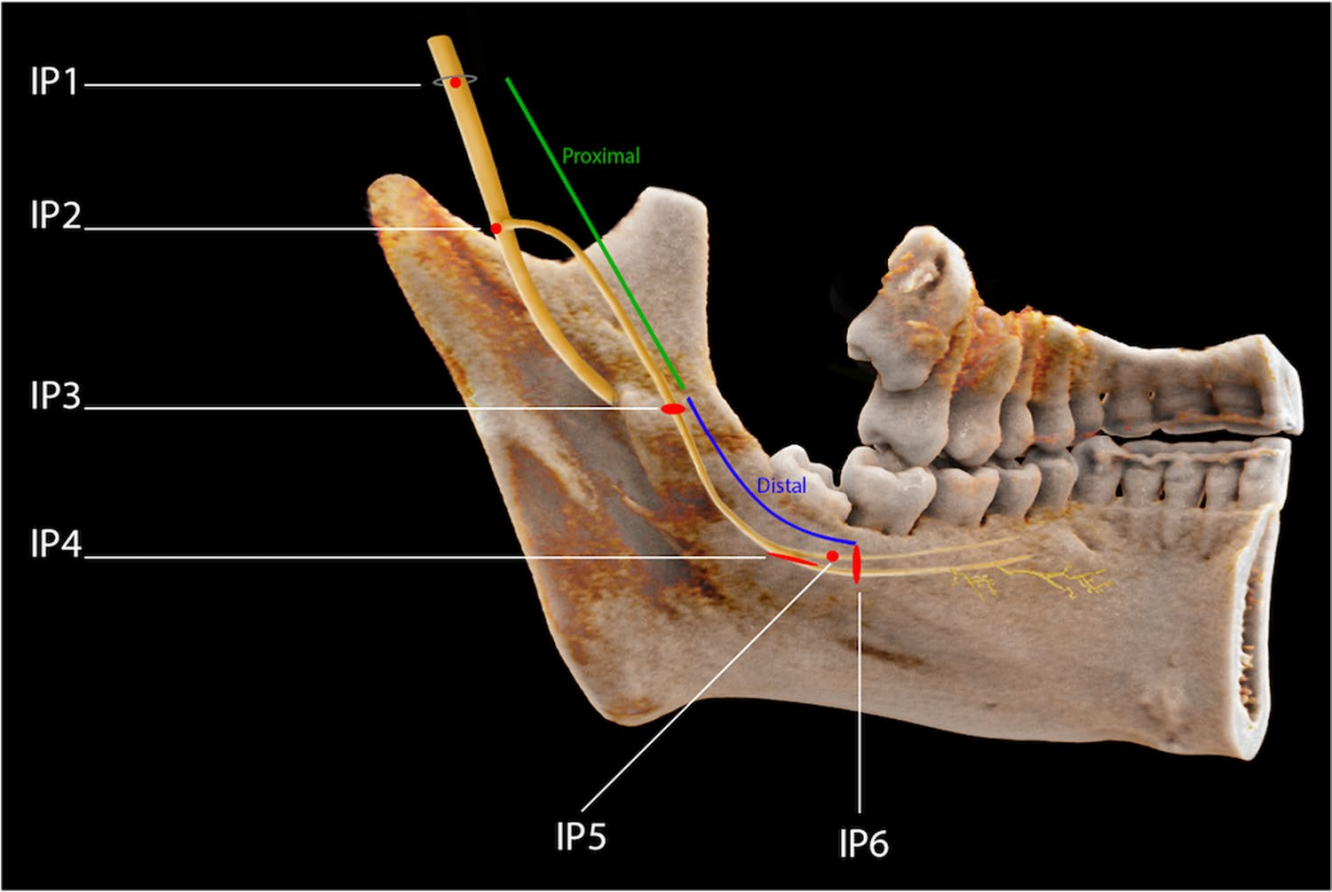
Photorealistic 3-dimensional (3D) visualization of a study participant’s cone beam computed tomography (CBCT) using cinematic rendering (CR). The division of the mandibular and lingual nerve (LN) into a proximal (IP1–IP3) and distal portion (IP3–IP6) with the 6 intermediate points (IP) is visualized. IP1, oval foramen; IP2, branching into the IAN and LN; IP3, LN starts running from laterally to medially; IP4, LN at the level of the third molar; IP5, gingival branch of the LN; IP6, LN at the height of the second molar

The MRI data was stored and evaluated in syngo.via (release VB30a, Siemens Healthineers, Erlangen, Germany) using a 2-MP high-resolution liquid–crystal display. The entire course of the LN was evaluated in randomized patient order by three readers with varying degrees of experience (reader A, attending board-certified neuroradiologist; reader B, attending board-certified oral surgeon; reader C, fourth-year dental medicine student). Before performing the evaluation, all three readers conducted a calibration session, in which some random cases were evaluated together to resolve any uncertainty. Intra-reader agreement was examined by having all readers repeat the readout after a time interval of minimum 2 weeks to avoid recall bias. The intra-reader agreement of reader C was selected to determine the expected lower bound for that particular non-neuroradiologist expert and investigate its applicability in the daily dental routine. All readers were blinded to the results of the other readers or their previous readout. Axial, coronal, and sagittal DESS multiplanar image reconstructions were available for quantitative and qualitative analysis. Qualitative analysis has been assessed using a 5-point Likert rating scale for overall technical image quality and a qualitative 4-point Likert scale for grading the artifacts. In addition, nerve continuity analysis and the focal detectability of the six intermediate points were evaluated (see Fig. 1 demonstrating nerve anatomy and intermediate points). As part of the quantitative analysis, the horizontal and vertical distance between LN and lingual cortical plate and the LN diameter at two defined locations were measured.

### Qualitative readout

First, the overall image quality was evaluated using a modified 5-point Likert scale, where motion artifacts, pulsation, and ghosting in the molar region were considered [22]: 0, excellent image quality with full diagnostic interpretability of the LN and surrounding tissue; 1, good image quality with full diagnostic interpretability of the LN and surrounding tissue; 2, acceptable image quality and diagnostic interpretability; 3, markedly reduced image quality and impaired diagnostic interpretability of the LN and tissue components; 4, severely reduced image quality, allowing no diagnostic interpretability of LN and/or surrounding tissue. Second, artifacts in the region of the first, second, and third molars were graded by a 4-point Likert scale: 0, absence of artifacts caused by dental restorations (none); 1, minor artifacts caused by dental restorations (low); 2, moderate artifacts caused by dental restorations (moderate); 3, massive artifacts caused by dental restorations (high).

The nerve continuity analysis was done according to Fujii et al. [17] by dividing the nerve into a proximal (from IP1 to IP3) and distal part (from IP3 to IP6) and checked for their continuous visibility using a modified 5-point scale: 4, excellent = both the proximal and distal portions of the nerve identified; 3, good = both the proximal and distal portions of the nerve identified but not continuous, with the distinction whether the interruption was proximal or distal; 2, fair = only the proximal portion of the nerve identified; 1, poor = only the proximal portion of the nerve identified but not continuous; 0, none = the nerve not identified (Fig. 1).

**Fig. 2:**
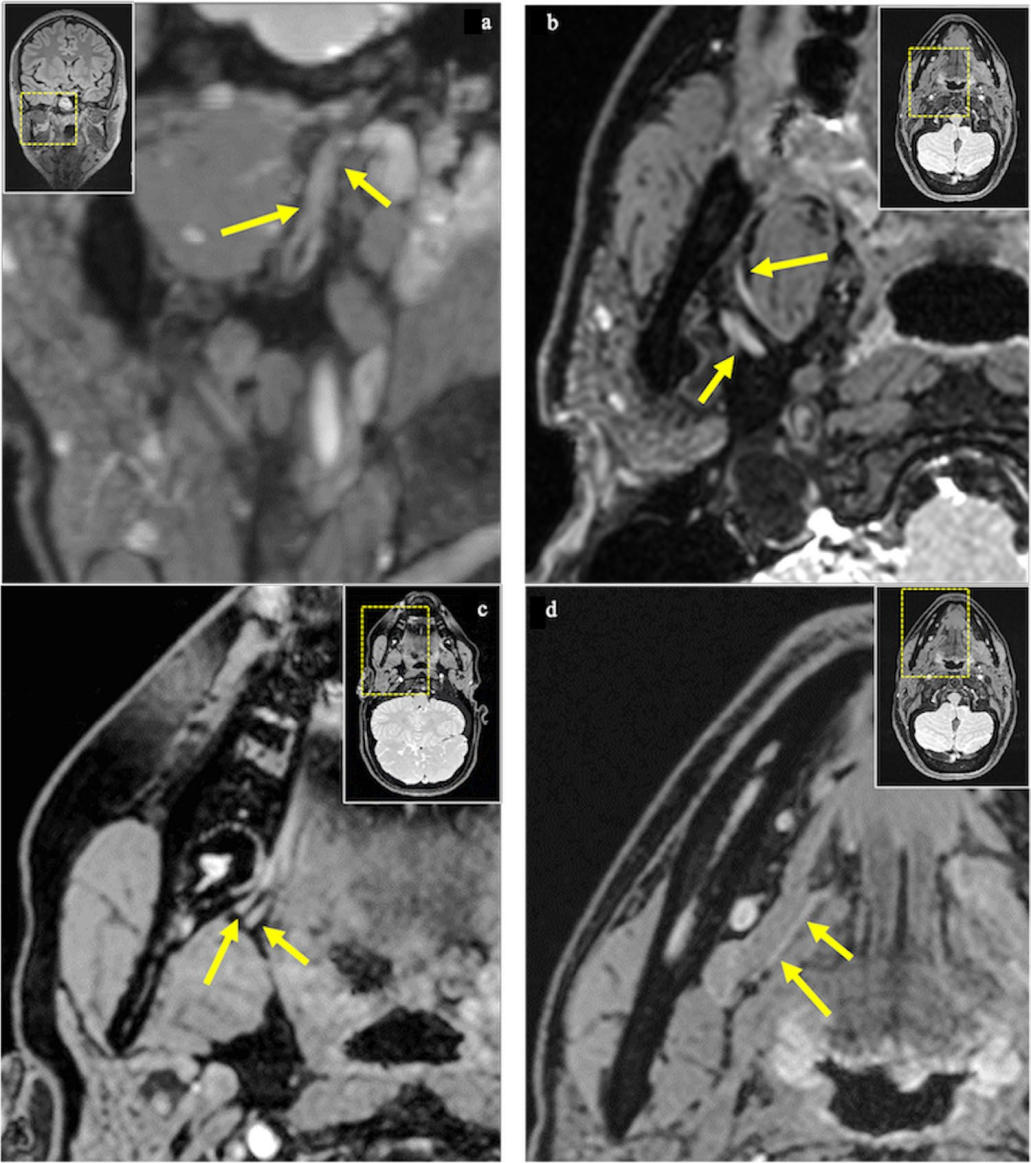
**a** Coronal and **b**–**d** axial reconstruction of the 3D-DESS sequence showing the intermediate points (IP). IP1 (**a**, short arrow): mandibular nerve within the oval foramen; IP2 (**a**, long arrow): branching of the inferior alveolar nerve (IAN) and the lingual nerve (LN). In the axial images, the IAN (**b**, short arrow) and the point in the course of the LN (**b**, long arrow) where it starts running from laterally to medially (IP3) are visible. IP4 (**c**, short arrow): LN at the third molar level; IP5 (**c**, long arrow): LN’s gingival branch. IP6 (**d**, long arrow) represents the LN at the second molar level and its further insertion into the tongue (**d**, short arrow)

Additionally, the LN was checked for the focal detectability of six intermediate points, which were selected in the extracranial course of the trigeminal nerve with special focus on the mandibular division (V3): the first point was the oval foramen (IP1), where the mandibular nerve (V3) descends and splits up in an anterior and posterior branch. The second intermediate point is localized at the point where the mandibular nerve branches into its various branches with a focus on the branching of the inferior alveolar nerve (IAN) and the LN (IP2). The LN, part of the posterior part, lying medial and in front of the IAN and beneath the lateral pterygoid muscle (LPM), is joined by the chorda tympani, a branch of the facial nerve (VII), and runs towards the medial pterygoid muscle (MPM). The nerve runs between the MPM and the ramus of the mandibula and crosses towards the side of the tongue. The point where the LN starts running from laterally to medially (IP3), the one and only intermediate point for the LN selected by Fujii et al., is the third intermediate point. The LN crosses the duct of the submandibular gland and runs in the third molar region where the fourth intermediate point was selected at the level of the third molar whether present or absent (IP4). The LN, characterized by its large anatomical variability, partially gives off a branch to the lingual gingiva that is referred to in the literature as “the gingival branch of the lingual nerve” [23] or the “collateral nerve twigs”(IP5) [24]. The last and sixth intermediate point is at the height of the tangent through the distal margin of the crown of the second molar (IP6) (Figs. 1 and 2).

The six intermediate points defined over the LN course were selected with special respect to the anatomical areas that are particularly vulnerable for the LN during third molar extraction treatments. Their detectability was checked using the following 4-point scale: 4, yes; 3, probably yes; 2, probably no; 1 no.

### Quantitative readout

The information about the horizontal and vertical distance between the LN and the alveolar ridge of the lingual cortical plate is important for surgical interventions on the third molars and was determined using axial and coronal images of the 3D-DESS sequence.

According to Burian et al. in 2019, the LN diameter was measured at a proximal and distal location to examine the preoperative nerve diameter assessment. The proximal measurement took place 2 cm caudal to the oval foramen in the infratemporal fossa and the distal measurement point was at the entrance of the LN into the pterygomandibular space opposite to the third molar region [18].

### Statistical analyses

Statistical analyses were performed using IBM SPSS Statistics software (version 25.0, IBM Corp. Armonk, NY, USA). The average of the nerve continuity analysis scores and intermediate point detectability values of each reader was calculated. In addition, the average scores of all three readers were calculated. A two-way mixed intraclass correlation coefficient (ICC) with a two-sided 95% confidence interval (CI) was used to calculate the absolute agreement of the intra- and interobserver variability of the nerve continuity analysis. The strength of agreement beyond chance obtained can be interpreted as follows: poor, < 0.5; moderate, 0.5–0.75; good, 0.75–0.9; excellent, > 0.9 [25]. The power analysis was estimated using existing literature [17]. Average score and inter- and intra-reader agreements regarding the qualitative assessment of image quality and measurements of artifacts were also analyzed. Regarding the six intermediate points’ focal detectability, intra- and interobserver agreement was reported as a percentage agreement. The nonparametric Mann–Whitney *U* test based on a two-tailed test with a significance of *p* < 0.05 was chosen to check whether there are significant differences between the diameter of the LN and the distance between the LN and the alveolar crest of the lingual cortical plate on the left and right side.

## Results

### Inter-reader and intra-reader agreement

The inter-reader and the intra-reader agreement between readers A, B, and C was moderate or good regarding the qualitative overall technical image quality (inter-reader ICC = 0.667, intra-reader ICC = 0.764; both *p* < 0.001) and the artifact severity (inter-reader ICC = 0.698, intra-reader ICC = 0.721; both *p* < 0.001).

The inter-reader and intra-reader agreement between the three readers regarding the nerve continuity analysis was good (inter-reader ICC = 0.764, intra-reader ICC = 0.749; both *p* < 0.001). Regarding the average percentage agreement in all intermediate points, the inter-reader agreement was 77% and the intra-reader agreement was 87% (Table 3).

**Table 3:** The intra- and interobserver variability of the 3 readers (A, B, and C). For the IP detectability analysis, the agreement was expressed as a percentage, while for the nerve continuity analysis, the absolute agreement using intraclass correlation coefficients (ICC) with a two-sided 95% confidence interval (CI) was assessed

Lingual nerve	A and B	B and C	C and A	A1 and A2	B1 and B2	C1 and C2	Average
Intermediate point 1	94.7%	94.7%	100%	100%	94.7%	100%	97.4%
Intermediate point 2	92.1%	86.8%	89.5%	97.3%	94.7%	94.7%	92.5%
Intermediate point 3	100%	100%	100%	100%	100%	100%	100%
Intermediate point 4	78.9%	89.5%	86.8%	92.1%	86.8%	92.1%	87.7%
Intermediate point 5	52.6%	73.7%	55.3%	78.9%	84.2%	73.7%	69.7%
Intermediate point 6	28.9%	34.2%	36.8%	71.1%	68.4%	39.5%	46.5%
Nerve Continuity Score (ICC (95% CI))	0.661 (0.44–0.81)	0.887 (0.79–0.94)	0.744 (0.61–0.88)	0.634 (0.38–0.8)	0.722 (0.53–0.85)	0.89 (0.79–0.94)	0.756

### Qualitative results

The average score of all readers for the overall image quality was 0.382 ± 0.7. Thus, the image quality was excellent with full diagnostic interpretability of the LN and surrounding tissue.

Artifacts caused by dental restoration in the molar region showed an average evaluation score of 0.256 ± 0.5. Thus, it can be stated that the DESS images showed ordinarily absence of artifacts caused by dental restorations.

The nerve continuity analysis demonstrated an average score of 3.3 ± 0.46 (good). In 35% of the cases, the entire course of the LN was fully continuously visible at least from IP1 to IP6, and in most cases until its insertion into the tongue. In 10% of the cases, the proximal part of the LN was not entirely continuously visible; in all cases, the interruption took place at IP2. In 60% of the cases, the distal part of the nerve was not entirely continuously visible; in most of these cases, the interruption was in the section where the LN runs forward to the distal margin of the second molar (Table 4).

**Table 4:** Average scores of the 3 readers (A, B, and C, 1 first readout and 2 s readout) evaluating the lingual nerve continuity by using a 5-point scale (4 = excellent; 3 = good; 2 = fair; 1 = poor; 0 = none) and the detectability of the intermediate points over the entire course of the lingual nerve by using a 4-point scale (4 = yes; 3 = probably yes; 2 = probably no; 1 = no)

Lingual nerve	Reader A1	Reader B1	Reader C1	Reader A2	Reader B2	Reader C2	Average
Intermediate point 1	4 (SD 0)	3.95 (SD 0.23)	4 (SD 0)	4 (SD 0)	4 (SD 0)	4 (SD 0)	3.99 (SD 0.04)
Intermediate point 2	3.95 (SD 0.23)	3.92 (SD 0.27)	3.95 (SD 0.23)	3.92 (SD 0.36)	3.95 (SD 0.23)	3.95 (SD 0.24)	3.94 (SD 0.26)
Intermediate point 3	4 (SD 0)	4 (SD 0)	4 (SD 0)	4 (SD 0)	4 (SD 0)	4 (SD 0)	4 (SD 0)
Intermediate point 4	3.79 (SD 0.53)	3.95 (SD 0.23)	3.95 (SD 0.23)	3.76 (SD 0.59)	3.82 (SD 0.46)	3.97 (SD 0.16)	3.87 (SD 0.37)
Intermediate point 5	3.07 (SD 1)	3.74 (SD 0.50)	3.71 (SD 0.77)	3.15 (SD 0.86)	3.68 (SD 0.53)	3.66 (SD 0.78)	3.5 (SD 0.74)
Intermediate point 6	2.45 (SD 1.33)	3.02 (SD 0.79)	3.05 (SD 1.04)	2.47 (SD 1.31)	3.05 (SD 0.8)	2.71 (SD 1.01)	2.79 (SD 1.05)
Nerve Continuity Score	3.37 (SD 0.49)	3.34 (SD 0.48)	3.32 (SD 0.47)	3.3 (SD 0.41)	3.26 (SD 0.45)	3.34 (SD 0.48)	3.32 (SD 0.46)

The average detectability score of all intermediate points was 3.7 ± 0.41. From IP1 to IP4, the detectability was excellent; IP5 had a slightly lower average score (3.5 ± 0.74) but could still be considered highly visible. In contrast to the first five intermediate points, IP6 was least detectable with an average score of 2.79 ± 1.05 (Table 4).

### Quantitative results

**Fig. 3:**
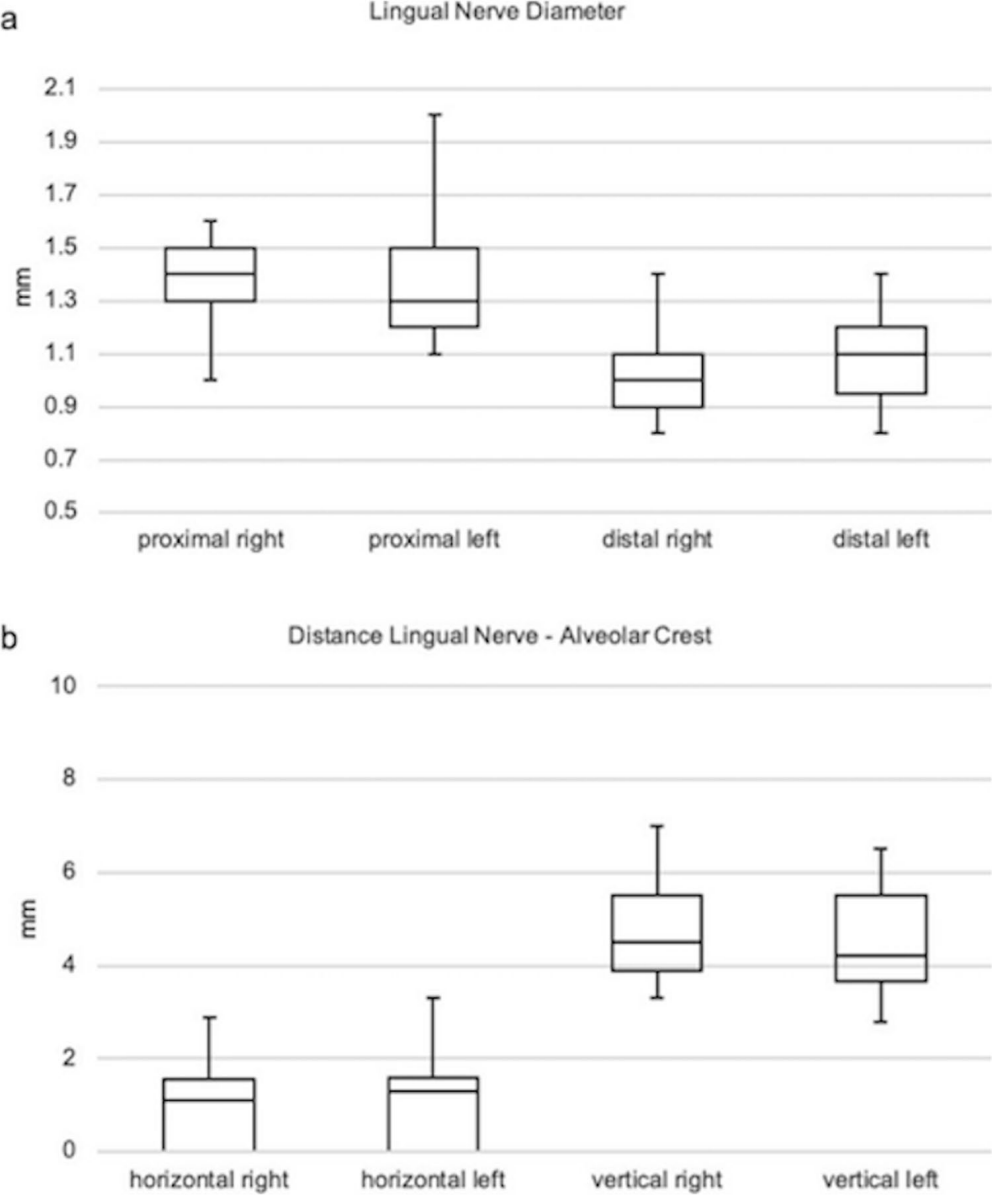
**a**, **b** Box plots of the lingual nerve diameter in proximal and distal locations (proximal right vs. left *p* = 0.454, distal right vs. left *p* = 0.402) and of the horizontal and vertical distances (horizontal right vs. left *p* = 0.435, vertical right vs. left *p* = 0.311) from the lingual nerve to the lingual cortical plate for the left and right side (Mann–Whitney *U* test *p* < 0.05). IQR, interquartile range; Q1, first quartile; Q3, third quartile. •: IQR (Q3–Q1), —: median

The mean horizontal distance between the LN and the alveolar crest of the lingual cortical plate was 1.05 ± 1.0 mm, and the mean vertical distance was 4.65 ± 1.2 mm. No significant differences were registered between both sides (horizontal right vs. left *p* = 0.435, vertical right vs. left *p* = 0.311) (Fig. 3; Table 5).

**Table 5:** The horizontal and vertical distance between the lingual nerve and lingual cortical plate was registered. No significant differences were found between the distances on the right side compared to the left side

Lingual nerve *N* = 38	Right	Left	Average
Alveolar crest – LN horizontal distance			
Mean ± standard deviation	0.91 (SD 1) mm	1.18 (SD 1.1) mm	1.05 (SD 1) mm
Median	1.1 mm	1.3 mm	1.2 mm
Lower range	0 mm	0 mm	0 mm
Upper range	2.9 mm	3.3 mm	3.1 mm
Mann–Whitney *U*, significantly different (*p* < 0.05)?	Right versus left No *p* value = 0.435		
Alveolar crest – LN vertical distance			
Mean ± standard deviation	4.87 (SD 1.2) mm	4.42 (SD 1.3) mm	4.65 (SD 1.2) mm
Median	4.6 mm	4.2 mm	4.4 mm
Lower range	3.3 mm	2.8 mm	3.1 mm
Upper range	7 mm	6.5 mm	6.8 mm
Mann–Whitney *U*, significantly different (*p* < 0.05)?	Right versus left No *p* value = 0.311		

The mean nerve diameter of the LN 2 cm caudal to the oval foramen was 1.42 ± 0.2 mm. The mean diameter at the entrance of the LN into the pterygomandibular space opposite to the third molar region was 1.06 ± 0.2 mm. No significant differences between the left and right sides were registered (proximal right vs. left *p* = 0.454, distal right vs. left *p* = 0.402) (Fig. 3; Table 6).

**Table 6:** Mean, median, and lower and upper range of the lingual nerve diameter in a proximal and distal location for left and right side. No significant differences could be detected regarding the measurements of both sides

Lingual nerve, *N* = 38	Proximal right	Proximal left	Average	Distal right	Distal left	Average
Mean	1.42 (SD 0.2) mm	1.41 (SD 0.3) mm	1.42 (SD 0.2) mm	1.03 (SD 0.2) mm	1.08 (SD 0.2) mm	1.06 (SD 0.2) mm
Median	1.4 mm	1.3 mm	1.4 mm	1 mm	1.1 mm	1.1 mm
Lower range	1 mm	1.1 mm	1.1 mm	0.8 mm	0.8 mm	0.8 mm
Upper range	1.6 mm	2 mm	1.8 mm	1.4 mm	1.4 mm	1.4 mm
Mann–Whitney *U*, significantly different (*p* < 0.05)?	Proximal right versus left No *p* = 0.454			Distal right versus left No *p* = 0.402		

## Discussion

This study investigated the visualization of the extracranial course of the LN with special respect to the anatomical relationship of the third molar teeth by using a 3D-DESS MRI sequence. This study’s results showed the feasibility of the focal and continuous detectability of the course of the LN with a high reproducibility. It was possible to generate reliable measurements of the nerve diameter and of the horizontal and vertical distance from the LN to the lingual cortical plate with a high concordance of both sides, which were consistent with the results of previous radiographic studies and anatomical cadaver studies [18, 26–28].

Despite challenges in detecting the extracranial peripheral nerves in clinical routine, several studies using different imaging modalities showed that direct visualization is feasible and accurate [14, 15, 17, 18]. Previous studies also proved that direct MRI visualization of the LN using the 3D-DESS sequence is possible [17, 18]. This sequence, currently successfully applied in musculoskeletal imaging and parotid tumor and facial nerve localization, with its fat-suppression technique, is well suited for depicting the LN due to the simultaneous application of the water excitation technique, providing uniform fat suppression and allowing the clear visualization of the LN independent of the surrounding fatty tissue [20].

In this study, it was possible to continuously visualize the entire LN course from the oval foramen to the distal margin of the second molar. Despite the small size, the ramification of the nerve, and the proximity to other anatomical structures, in some cases, it was possible to visualize the nerve until its insertion into the tongue. In almost all cases (92%), it was possible to display the LN continuously at the level of the third molar; meanwhile, the continuous visibility got lost in 60% of the cases in the area where the LN runs forward to the second molar and further. The fact of not being able to display the LN at this level of its course could be explained by various causes, such as the anatomy of the nerve, the imaging technique, or the readers. The achieved inter-reader agreement showed that despite the great differences in the readers’ experience, this diagnostic method is suitable with the potential to be established in clinical routine in the future. However, prospective studies are required to assess the therapeutic and clinical impact in patients undergoing third molar removal having preoperative MRI.

Regarding the quantitative parameters, we got reproducible nerve diameter measurements for the proximal and distal location showing no significant differences between both sides. Burian et al.’s MRI study presented slightly higher proximal and distal median (proximal + 0.2 mm, distal + 0.3 mm) values than this study; regarding the upper and lower range proximal and distal, the values obtained showed high concordance [18]. Considering that no significant differences were found in diameter inter-side, these values can be seen as comparable, as minimal differences may have occurred due to anatomical variation or to the resolution of the DESS images. The mean horizontal distance from the LN to the lingual cortical plate and lingual crest of the mandible using MRI visualization was 1.05 ± 1.0 mm and the mean vertical distance was 4.65 ± 1.2 mm. These parameters important for performing third molar extraction with minimal risk to LN damage were consistent with some previously performed anatomical cadaver studies [26–28]. Other studies demonstrated about 3 to 8 mm higher mean horizontal, and about 2 to 3 mm higher mean vertical distances [29, 30]. However, this is probably based on the high intervariation of the LN in the third molar region.

To date, the cost-effectiveness is not given yet, as dental MRI imaging is still not considered a standard diagnostic procedure in the context of preoperative radiological assessment prior to third molar extraction treatment. However, the constantly increasing number of MRI reports in the dental field confirms the importance and perspectives that are opening up due to the targeted use of this radiation-free imaging modality. Further refinement of this MRI protocol and possible application in MR neurography using high-field-strength MRI could allow the differentiation of the skull base from extracranial nerve segments in T2-weighted images with high soft tissue contrast and homogenous fat, arterial, and venous suppression. This accurate visualization might be useful in various other surgical procedures, such as dental implant placement, mandibular split osteotomy, or detection of post-traumatic trigeminal lesions [31, 32]. Therefore, MRI imaging might provide benefits for the patient and the clinician in challenging cases.

Several limitations in this study should be mentioned. Firstly, there is a methodical limitation because of the small cohort size. Therefore, it is challenging to make generally valid statements; larger cohorts and further investigations are needed to confirm these qualitative and quantitative measurements with higher certainty, allowing ideal sample size calculation. Secondly, the results of the image quality and artifact susceptibility evaluation should be interpreted with caution as adjacent implants or metallic restorations were considered as exclusion criteria. Nevertheless, the literature indicates various other sources of artifacts: stainless-steel brackets, NiTi archwires, zirconia reconstructions, fillings with glass ionomer cement, composite resins, or even the use of zinc phosphate–based cement can trigger artifacts in MRI, too. Therefore, to achieve standardized, reliable data, even though metallic artifacts were per design excluded, the influence of motion and other non-metallic artifacts sources could still be investigated here. Thirdly, there is the anatomical course of the LN with its variations, especially in the third molar region, which makes the evaluation more difficult. To be more precise, this concerns its relationship to the alveolar ridge of the lingual cortical plate, whether there is a gingival branch or not, the place where the looping around the submandibular duct takes place and the anatomic proximity to other structures and their arrangement. In addition, the small size of the nerve in the investigated area: the finer the structure, the more difficult it is to distinguish the nerve from the surrounding and partially overlapping anatomical structures. Despite this fact, it was possible to visualize the nerve until its insertion into the tongue. For better visualization, refinement of the sequence should be part of future investigations. Fourthly, most of the participants were young, so they hardly showed any artifacts caused by dental restorations. Future investigations should also examine the influence of these factors on the detectability of the LN. Therefore, the MRI sequence’s refinement should also deal with the minimization or even elimination of this kind of artifacts, maybe using zero TE or ultra-short TE methods. Considering the MRI artifact susceptibility of the head and neck area, some features and modifications of the DESS sequence were beneficial for the optimization process in MRI data acquisition; e.g., the double-echo acquisition of the DESS sequence provides, in a simplified view, good T2 contrast from one echo and low artifacts from the other. Furthermore, the high contrast diminishes intravoxel dephasing artifacts due to magnetic field inhomogeneities. Finally, the omission of grappa acceleration reduced the noise at the center of the FOV and explained our DESS implementation’s relatively long scan time. Nevertheless, future studies should focus on these issues—possibly using different readout trajectories—to further improve MRI of the oral cavity. In addition, further research could be conducted to optimize image quality with respect to the contrast to noise ratio of other tissues than the LN, as this appears to be relatively low for the evaluated DESS sequence.

Modern surgical management of third molar extractions aims to avoid lingual flap detachment to protect the LN in the retromolar space [9]. Therefore, detailed knowledge of the LN course, its diameter, and its distance to the lingual cortical plate in the third molar region potentially reduces the risk for the surgeon to a minimum in cases where the surgical area has to be extended into the lingual area, due to the position of the third molar. As with most studies available in the literature, this is an exploratory study testing the feasibility of nerve visualization. Based on these data confirming the visualization of the LN and further depicting its continuous course and considering the large heterogeneity in the literature regarding scan parameters and the use of different magnetic field strengths, further studies should be conducted, including randomized control trials examining the evidence of the individual MRI protocol in a given medical condition, to provide an evidence-based understanding of its use and propose further information on how preoperative radiological assessment might influence the clinical outcome. Although MRI imaging is not yet an integral part of everyday dentistry, a close positional relationship to adjacent anatomical structures, short MRI examination times, and high detectability for the entire continuous LN course with high concordance by readers with different training levels might justify the use of it. After being validated in larger cohort groups and pathological cases, the targeted use of this MRI sequence might have the potential to be integrated in clinical routine and thus avoid the unpleasant postoperative complications associated with LN damage for the patients.

## Conclusion

The implemented MRI sequence allowed for mostly continuous visualization of the entire course of the lingual nerve from the oval foramen to the third molar region with high confidence and reproducibility. This enables a preoperative clarification of the LN position in patients undergoing surgical third molar extraction treatment. The use of this MRI technology generates reproducible information about the LN diameter and its horizontal and vertical distance to the lingual cortical plate. This information could prevent complications in various oral surgical procedures.

## Supplementary Information

Below is the link to the electronic supplementary material.

Supplementary file1 (PDF 103 KB)
