# Supplementary material for: Preoperative visualization of the lingual nerve by 3D double-echo steady-state MRI in surgical third molar extraction treatment
Source: Clin Oral Investig. 2021 Sep 29;26(2):2043–53. doi: 10.1007/s00784-021-04185-z (PMC8816737; doi:10.1007/s00784-021-04185-z)
Supplement: Supplementary file 1 — Supplementary file1 (PDF 103 KB) [file 784_2021_4185_MOESM1_ESM.pdf]

\\Neuro_Forschung\MR Physics\cooperation\MKGPat012\dess_0.75iso_flowComp
TA: 12:24 PM: FIX Voxel size: 0.4×0.4×0.8 mmPAT: Off Rel. SNR: 1.00 : de_rr

Properties

Prio recon	Off
Load images to viewer	On
Inline movie	Off
Auto store images	On
Load images to stamp segments	Off
Load images to graphic segments	Off
Auto open inline display	Off
Auto close inline display	Off
Start measurement without further preparation	Off
Wait for user to start	Off
Start measurements	Single measurement

Routine

Slab group	1
Slabs	1
Dist. factor	20 %
Position	L0.0 P21.2 H23.4 mm
Orientation	T > C32.6 > S-2.7
Phase enc. dir.	R >> L
AutoAlign	---
Phase oversampling	0 %
Slice oversampling	100.0 %
Slices per slab	104
FoV read	242 mm
FoV phase	100.0 %
Slice thickness	0.75 mm
TR	11.16 ms
TE	4.21 ms
Averages	1
Filter	Prescan Normalize
Coil elements	HC3-7;NC1,2

Contrast - Common

TR	11.16 ms
TE	4.21 ms
Flip angle	30 deg
Fat suppr.	Water excit. normal

Contrast - Dynamic

Averages	1
Averaging mode	Short term
Reconstruction	Magnitude
Measurements	1
Multiple series	Each measurement

Resolution - Common

FoV read	242 mm
FoV phase	100.0 %
Slice thickness	0.75 mm
Base resolution	320
Phase resolution	100 %
Slice resolution	100 %
Phase partial Fourier	Off
Slice partial Fourier	Off
Interpolation	On

Resolution - iPAT

PAT mode	None

Resolution - Filter Image

Image Filter	Off
Distortion Corr.	Off
Prescan Normalize	On
Unfiltered images	Off
Normalize	Off
B1 filter	Off

Resolution - Filter Rawdata

Raw filter	Off
Elliptical filter	Off

Geometry - Common

Slab group	1
Slabs	1
Dist. factor	20 %
Position	L0.0 P21.2 H23.4 mm
Orientation	T > C32.6 > S-2.7
Phase enc. dir.	R >> L
Slice oversampling	100.0 %
Slices per slab	104
FoV read	242 mm
FoV phase	100.0 %
Slice thickness	0.75 mm
TR	11.16 ms
Multi-slice mode	Sequential
Series	Interleaved

Geometry - AutoAlign

Slab group	1
Position	L0.0 P21.2 H23.4 mm
Orientation	T > C32.6 > S-2.7
Phase enc. dir.	R >> L
AutoAlign	---
Initial Position	L0.0 P21.2 H23.4
L	0.0 mm
P	21.2 mm
H	23.4 mm
Initial Rotation	90.00 deg
Initial Orientation	T > C
T > C	32.6
> S	-2.7

Geometry - Tim Planning Suite

Set-n-Go Protocol	Off
Table position	H
Table position	0 mm
Inline Composing	Off

System - Miscellaneous

Positioning mode	FIX
Table position	H
Table position	0 mm
MSMA	S - C - T
Sagittal	R >> L
Coronal	A >> P
Transversal	F >> H
Coil Combine Mode	Adaptive Combine
Save uncombined	Off
Matrix Optimization	Off
AutoAlign	---

System - Miscellaneous

Coil Select Mode	On - AutoCoilSelect

System - Adjustments

B0 Shim mode	Standard Neck
B1 Shim mode	Patient-specific
Adjust with body coil	Off
Confirm freq. adjustment	Off
Assume Dominant Fat	Off
Assume Silicone	Off
Adjustment Tolerance	Auto

System - Adjust Volume

Position	L0.0 P21.2 H23.4 mm
Orientation	T > C32.6 > S-2.7
Rotation	180.00 deg
A >> P	61 mm
R >> L	242 mm
F >> H	78 mm
Reset	Off

System - pTx Volumes

B1 Shim mode	Patient-specific
Excitation	Slab-sel.

System - Tx/Rx

Frequency 1H	123.161543 MHz
Correction factor	1
Gain	Low
Img. Scale Cor.	1.000
Reset	Off
? Ref. amplitude 1H	0.000 V

Physio - PACE

Resp. control	Off

Inline - Common

Subtract	Off
Measurements	1
StdDev	Off
Save original images	On

Inline - MIP

MIP-Sag	Off
MIP-Cor	Off
MIP-Tra	Off
MIP-Time	Off
Save original images	On

Inline - Composing

Inline Composing	Off
Distortion Corr.	Off

Sequence - Part 1

Introduction	On
Dimension	3D
Elliptical scanning	Off
Asymmetric echo	Off
Flow comp.	Read
Multi-slice mode	Sequential
Bandwidth	355 Hz/Px

Sequence - Part 2

Segments	1

Sequence - Part 2

RF pulse type	Fast
Gradient mode	Fast
Excitation	Slab-sel.

Sequence - Assistant

Mode	Off
